# Synthesis, Sintering, and Characterization of Composites

**DOI:** 10.3390/ma19061130

**Published:** 2026-03-14

**Authors:** Biao Guo, Xiaonan Mu, Hongmei Zhang, Xingwang Cheng

**Affiliations:** National Key Laboratory of Science and Technology on Materials Under Shock and Impact, Beijing 100081, China

## Introduction

Composites, renowned for their lightweight nature, high strength, high specific modulus, high temperature resistance, and excellent wear resistance, have become indispensable key materials in fields such as aerospace, rail transportation, defense, and energy [1,2,3]. In recent years, the field of composites has developed rapidly, with emerging frontiers continuously expanding. They have begun to partially replace traditional structural materials, finding applications in extreme service environments characterized by ultra-high temperatures and ultra-high strain rates [4,5,6,7,8]. Composites can be classified by their matrix type into metal matrix composites (MMCs), polymer matrix composites (PMCs), ceramic matrix composites (CMCs), and carbon-based composites, among others. In terms of spatial architecture design, they can be configured as network, layered, island, or gradient structures [9,10,11,12]. Reinforcements include ceramic particles, whiskers, fibers, or other nanomaterials, such as zero-dimensional (e.g., nanodiamonds), one-dimensional (e.g., carbon nanotubes), and two-dimensional nanomaterials (e.g., graphene) [13,14,15]. Their performance is fundamentally determined by the complex interplay between the synthesis route, the densification process, and the resulting microstructural characteristics. Understanding the “process-structure-property” paradigm has become crucial for designing composites with tailored functionalities. This Special Issue (SI), “Synthesis, Sintering, and Characterization of Composites”, presents innovative research findings on several typical classes of composites, with a particular focus on innovative design in synthesis processes and microstructures, as well as the exploration of underlying mechanisms. This Editorial summarizes the five publications (four research articles and one review article) included in this SI.

Powder metallurgy is both a traditional and a dynamic advanced material forming technology. It offers mature techniques for preparing MMCs based on metals like titanium, copper, and aluminum, making it a preferred method for composite fabrication [16,17,18]. The process typically involves designing reinforced/metal composite powders, followed by consolidation or thermo-mechanical deformation, often requiring specialized conditions like high temperature and high pressure. Sintering is a primary consolidation technique, including methods such as spark plasma sintering (SPS), hot pressing (HP), hot isostatic pressing (HIP), and liquid phase sintering (LPS) [19,20,21,22]. How to precisely control the microstructure through reinforcement design, architectural design, and optimization of sintering processes to achieve tailored performance has always been a core challenge in this field [23,24,25].

Deepening the application of powder metallurgy techniques can further enhance the properties of MMCs to meet diverse material requirements. Pavkov et al. [26] utilized liquid phase sintering to introduce an environmentally friendly andesite basalt glass phase into a 316 L stainless steel matrix. Their work systematically reveals the influence of the glass phase content on the densification process, microstructural evolution, and mechanical properties of the composite.

Building upon existing sintering technologies to push the performance boundaries of MMCs, leveraging layered or gradient architectural design enables the precise construction of multi-scale structures and the cross-scale synergistic regulation of heterogeneous materials, synergistically enhancing strength and plasticity [27,28,29]. Duan et al. [30] employed field-assisted sintering technology (FAST) to successfully fabricate a bilayer composite plate, consisting of a high-strength-and-toughness “nacre-like” structured graphene nanoplatelet (GNP)/TC4 composite layer and a highly ductile TC4 layer. They conducted an in-depth analysis on the failure mechanism of this hierarchical heterogeneous structure under high-strain-rate impact. The study found that the hard layer dissipates energy by inducing multiple cracks, while the soft layer effectively arrests crack propagation, significantly enhancing the overall ballistic performance (V50 value). This provides a new paradigm for the integrated “processing-structure-property” design of high-performance MMCs intended for extreme impact service environments.

The nature of the interface in composites is critical in determining their integrity, reliability and mechanical properties [31]. The review by Zhu et al. [32] focuses on basalt fiber-reinforced polymer composites. It systematically summarizes various non-destructive surface modification techniques, such as treatment with silane coupling agents, rare-earth coupling agents, and plasma. It details how these methods introduce active functional groups, increase surface roughness, or construct nano-reinforced interlayers to effectively enhance the interfacial bonding strength between the fiber and the matrix.

Thermal spraying technology is an effective method for preparing functional ceramic matrix composites. Gadzhiev et al. [33] successfully used low-pressure plasma to melt and deposit β-Ga_2_O_3_ powder, forming a ceramic body with good crystallinity and luminescent properties. Their study accurately describes the heating and acceleration behavior of powder particles in the plasma jet, revealing the influence of process parameters on the phase composition, microstructure and photoluminescence properties of the final product.

In the realm of amorphous alloy composites designed for high-strain-rate impact environments, Wang et al. [34] synthesized a Ti_42.3_Zr_28_Cu_8.3_Nb_4.7_Ni_1.7_Be_15_ (at%) composite via vacuum melting. They conducted a detailed investigation into the synergistic deformation mechanism of this in situ formed metastable β-phase reinforced bulk metallic glass composite under tensile load. Beyond traditional shear banding, they observed localized amorphization and a stress-induced phase transformation from BCC β-Ti to HCP α-Ti at the crystalline/amorphous interface. This localized structural transition effectively relaxes stress concentration and leads to serrated flow behavior in the stress–strain curve, imparting excellent work-hardening capacity and tensile plasticity to the material.

Composites are a crucial material foundation supporting the development of advanced equipment manufacturing, and continuous performance improvements directly drive the evolution of next-generation high-end equipment [35,36]. In composite research and development, synthesis preparation, process control, and detailed characterization form the tightly interconnected core “process-structure-property” chain. The synergy among these three elements dictates the depth of material innovation and the breadth of engineering application. Optimal design must systematically consider how process parameters influence the evolution path of the microstructure and ultimately determine the macroscopic service behavior of the material [37]. Currently, the expansion of additive manufacturing technologies into metal, ceramic, and polymer matrix composites [38,39,40,41], the emergence of cold sintering processes for ceramic–polymer integration [42,43], and the maturation of energy-efficient methods like microwave sintering [44,45] are significantly broadening the design freedom and processing window for advanced composites. Simultaneously, high-throughput characterization methods integrated with machine learning are providing quantitative support for deeply deciphering the intrinsic correlations within the “process-structure-property” paradigm. Future breakthroughs in composites will rely on the deep integration and closed-loop synergy of the aforementioned fields [46]. The trend points towards building an intelligent closed-loop ecosystem driven by advanced manufacturing, big data technologies, and AI foundation models [47,48,49]. In this vision, leveraging massive experimental and service data, large models can inversely design optimal architectures. Advanced manufacturing then precisely realizes these designed architectures through controlled processing. Meanwhile, big data technologies continuously learn from the entire manufacturing and service lifecycle, feeding back information to iteratively optimize the models. This will ultimately propel composite materials research and development from the traditional “trial-and-error” paradigm into a new era of “data-driven intelligent design and precision manufacturing” [50], meeting the increasingly demanding service requirements of aerospace, energy, and other critical sectors.
